# Postoperative free flap monitoring in reconstructive surgery—man or machine?

**DOI:** 10.3389/fsurg.2023.1130566

**Published:** 2023-02-22

**Authors:** Samuel Knoedler, Cosima C. Hoch, Lioba Huelsboemer, Leonard Knoedler, Viola A. Stögner, Bohdan Pomahac, Martin Kauke-Navarro, David Colen

**Affiliations:** ^1^ Department of Plastic, Hand and Reconstructive Surgery, University Hospital Regensburg, Regensburg, Germany; ^2^ Department of Surgery, Division of Plastic Surgery, Yale School of Medicine, Yale New Haven Hospital, New Haven, CT, United States; ^3^ Department of Otolaryngology, Head and Neck Surgery, Rechts der Isar Hospital, Technical University Munich, Munich, Germany

**Keywords:** flap monitoring, free tissue transfer, microvascular reconstruction, microsurgery, free flap, reconstructive surgery

## Abstract

Free tissue transfer is widely used for the reconstruction of complex tissue defects. The survival of free flaps depends on the patency and integrity of the microvascular anastomosis. Accordingly, the early detection of vascular comprise and prompt intervention are indispensable to increase flap survival rates. Such monitoring strategies are commonly integrated into the perioperative algorithm, with clinical examination still being considered the gold standard for routine free flap monitoring. Despite its widespread acceptance as state of the art, the clinical examination also has its pitfalls, such as the limited applicability in buried flaps and the risk of poor interrater agreement due to inconsistent flap (failure) appearances. To compensate for these shortcomings, a plethora of alternative monitoring tools have been proposed in recent years, each of them with inherent strengths and limitations. Given the ongoing demographic change, the number of older patients requiring free flap reconstruction, e.g., after cancer resection, is rising. Yet, age-related morphologic changes may complicate the free flap evaluation in elderly patients and delay the prompt detection of clinical signs of flap compromise. In this review, we provide an overview of currently available and employed methods for free flap monitoring, with a special focus on elderly patients and how senescence may impact standard free flap monitoring strategies.

## 1. Introduction

Free tissue transfer (free flap; FF) represents a routine option for soft or composite tissue reconstruction, with the goal of providing durable wound coverage, improving aesthetic appearance, and restoring functional deficits. FFs offer a variety of tissue types, such as skin, muscle, bone, nerves, or a combination thereof and allow the reconstruction of sizeable tissue defects. The indications for FF surgery, therefore, are broadly defined, ranging from congenital anomalies through burn injuries and cancerous lesions to severe trauma-related tissue defects (1). Given the narrow diameter of FF vasculature, the blood supply is ensured *via* microvascular anastomosis between the donor and recipient vessel (2, 3). With the integrity and patency of this small-caliber anastomosis being crucial for the FF survival, arterial and venous thromboses are considered the most common reasons for FF failure (4–6). Vascular comprise typically manifests within 48 h after surgery (7, 8). Recent studies have demonstrated that, in these cases of vascular occlusion, the likelihood of FF salvage primarily depends on early diagnosis and return to the operating room. Briefly, the longer it takes to detect vascular incidents, the poorer the overall chances for FF survival (4, 9–11). Therefore, immediate detection and prompt intervention are essential for FF salvage, rendering close-knit postoperative FF monitoring indispensable.

**Figure 1:**
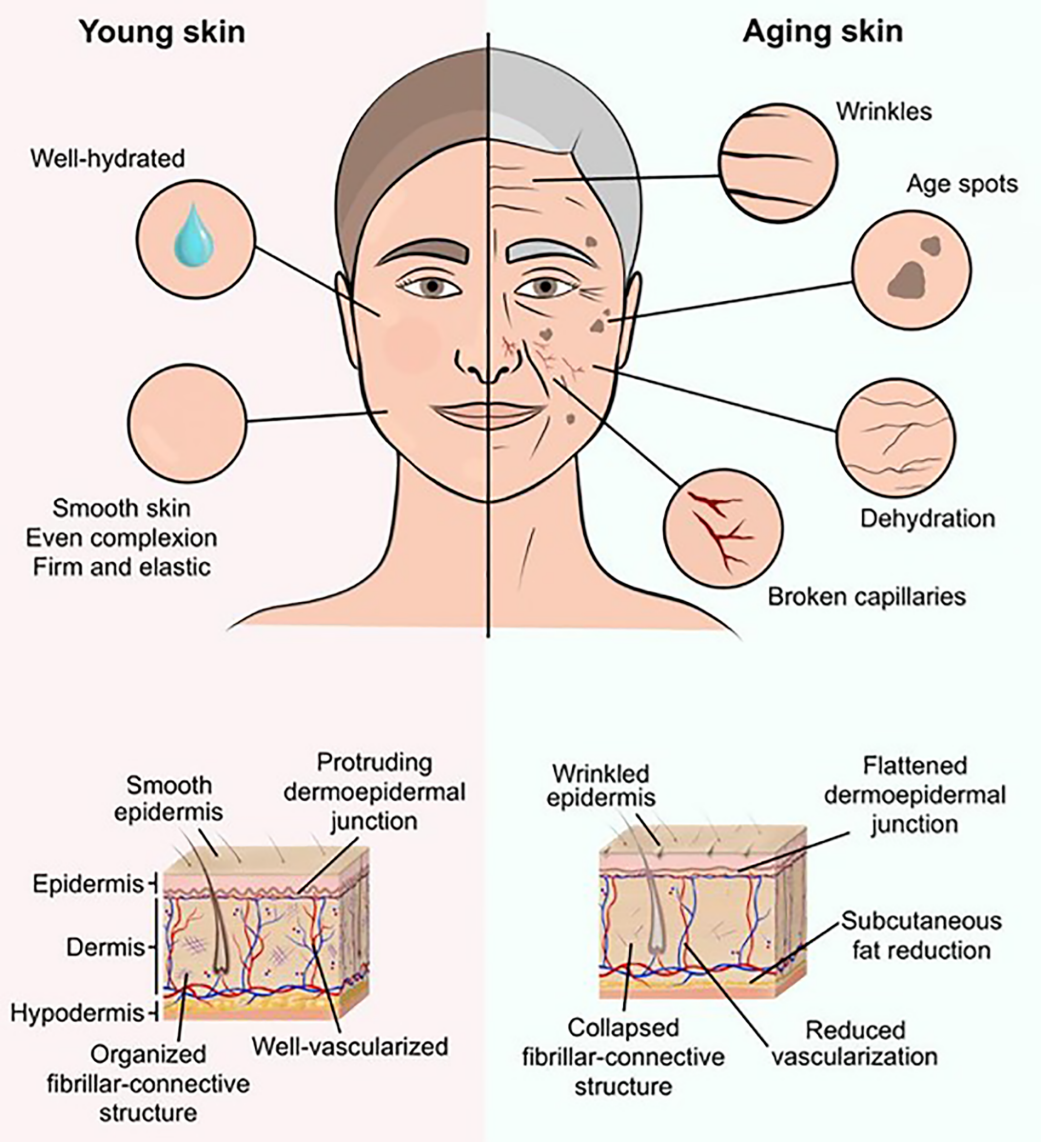
Illustrative comparison between young and aging skin. Age-related skin changes can manifest in various ways, thereby complicating free flap evaluation and concealing common signs of free flap failure. Externally, deep wrinkles, dark-pigmented age spots, and superficial vascular drawings reflect the senescence of the skin structures. On the histological level, the dermo-epidermal junction flattens and the fibrillar-connective structure collapses. Due to a progressive subcutaneous fat reduction, decrease in water content, and loss of elasticity the impression of youthful smooth skin vanishes over time.

With the ongoing demographic change, the number of older FF candidates is on the rise (12). Generally, as patients age, their cardiovascular stability, immune system competence, and wound healing capacities decrease. This age-related decline in health exacerbates surgical vulnerability and predisposes to postoperative complications. Accordingly, close-knit FF monitoring in elderly patients is of paramount importance, ensuring the early detection of adverse events and allowing timely intervention. Such surveillance strategy may, therefore, also prevent the need for reoperation and avert repeated perioperative stress and risks for susceptible elderly patients. However, age-specific skin changes, such as the loss of elasticity, the decrease in water content, and the fragility of the vascular bed (with subsequent vulnerability to hematoma), may complicate FF assessment and delay the prompt detection of flap failure (Figure 1) (13–16). In addition, advanced age has been associated with an increased risk of morbidity and mortality following FF surgery (17–19). Consequently, special attention must be paid to this vulnerable and frail patient population in the perioperative FF setting.

To date, the gold standard of FF monitoring includes clinical examination (CE; i.e., flap color, capillary refill, tissue turgor, temperature) and handheld acoustic Doppler sonography (ADS) (20, 21). However, an accurate and reliable CE requires experience and well-trained eyes. Such expertise is all the more important considering the diversity of warning symptoms and FF failure manifestations among different genders, ethnicities, and age groups. For example, assessment of skin changes (such as erythema) in Black patients may be less reliable (22). As a result, these methods of FF surveillance remain a logistical challenge for small, limited-hour residency programs and private practices as well as maximum-care hospitals with a high rate of emergencies and residents assisting in the operating room (23). In addition, CE and ADS may not be used for the monitoring of poorly accessible and deeply-buried flaps (24).

To address the shortcomings of these conventional strategies, there is an increasing interest in alternative techniques that may complement or even replace CE and ADS. This review aims to provide an overview of currently available and employed techniques for FF monitoring, with a special focus on the care of older patients. To this end, we discuss promising strategies through a medical-technical lens, linking clinical findings with relevant biophysical research. This may help physicians to upgrade their armamentarium of FF monitoring techniques and leverage their specific benefits.

## 2. Standard of care in free flap monitoring

**Figure 2:**
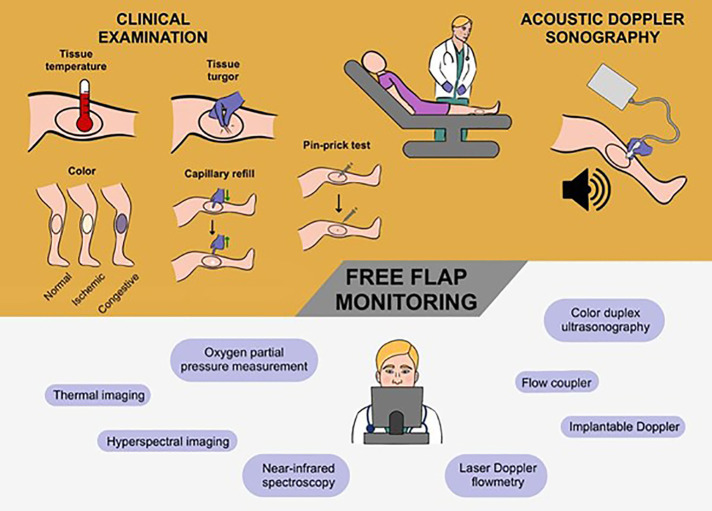
The current gold standard and novel techniques in the postoperative monitoring of free flaps. To date, the combination of clinical examination and acoustic Doppler sonography is still considered the state of art to monitor free flaps. More specifically, the attending physician examines the free flap serially evaluating the flap temperature, turgor, color, and capillary refill. Typically, this cornerstone of free flap assessment is supported by acoustic Doppler sonography as an instrument to sound the blood flow and velocity. Accordingly, the gold standard is based on a hands-on approach with the active-practical involvement of the physician at periodic intervals. In recent years, a broad spectrum of novel technologies has been proposed to facilitate and refine free flap monitoring. These high-tech methods range from the well-known color duplex sonography through implantable Doppler systems to hyperspectral and thermal imaging. In contrast to the conventional approach, this new generation of monitoring tools relies on digital (remote) equipment that allows the biophysical flap condition to be assessed in a technologically advanced and automatic-computerized form.

CE and handheld ADS still represent the standard of care in FF monitoring (25). Recently, novel modalities emerged as alternatives to these two well-established techniques (Figure 2). Yet, any of these techniques have inherent strengths and limitations. In the following, we summarize the most frequently used techniques in FF monitoring and outline their applicability (Table 1).

**Table 1:** Detailed overview of the free flap monitoring methods. Each of the presented methods has its inherent strengths and limitations, with the clinical examination in combination with acoustic Doppler sonography still being universally accepted as the gold standard in the postoperative monitoring of free flaps. While modern technologies may offer unique benefits for both patients and physicians, their pitfalls thwart widespread clinical implementation. This holds also true for the arguable field of free flap surveillance in elderly patients: Due to the lack of well-established and age-appropriate methods/tools, the conventional approach continues to be applied.

Monitoring Method	Strengths	Limitations	Applicability/Suitability in Elderly Patients
Clinical Examination	Noninvasive Low cost Quick assessment	Limited use in buried flaps without skin island Dependence on a light source Lack of standardization Expertise and experience required	Challenging due to interindividual skin/flap appearance and lacking standardized protocols
Acoustic Doppler Sonography	Noninvasive Simple to use Low cost Quick assessment	Typically not applicable for buried flaps Lack of quantitative measurement Noncontinuous Expertise and experience required	No definitive conclusions; age may be considered a potential confounder
Color Duplex Ultrasonography	Noninvasive Use in buried flaps Quantifications of vessel inflow and outflow	Expertise and experience required Expensive Noncontinuous	Lack of robust data regarding postoperative free flap monitoring
Flow Coupler	Simple to use Easy handling and simple setup Use in buried flaps Continuous	Invasive Implantable probe needs to be removed, (possible port of entry for infectious agents)	Might be beneficial, future large scale-studies are needed
Implantable Doppler	Simple to use Easy handling and simple setup Use in buried flaps Continuous	Invasive Expensive Implantable probe needs to be removed (possible port of entry for infectious agents)	Might be beneficial, future large scale-studies are needed
Laser Doppler Flowmetry	Noninvasive Continuous Arterial and venous occlusion differentiation	Expensive Lack of quantitative measure User training and experience required	Blood flow parameters may differ among elderly patients; standardized norm values are lacking
Near-Infrared Spectroscopy	Noninvasive Continuous Real-time measurement Simple to use Remote monitoring possible	Expensive Regional oxygen saturation might be influenced by internal and external variables Patient mobility can influence signal quality Requires skin island and taping of large probe that can limit clinical assessment of flap	Might be beneficial, future large scale-studies are needed
Hyperspectral Imaging	Noninvasive Quick assessment Ability to differentiate arterial and venous occlusion	Noncontinuous Expensive Sensitivity to small movement Lack of large-scale validation studies Reliance on ambient lighting	Resting posture might be problematic in elderly patients (e.g., due to tremor and restlessness)
Thermal Imaging	Noninvasive	Unclear, future studies are needed	Future large scale-studies are needed
Oxygen Partial Pressure Measurement	Continuous	Invasive Single-point measurement	Might be beneficial, future large scale-studies are needed

### 2.1. Clinical examination

Generally, a holistic overview of the patient's health is fundamental when evaluating the FF postoperatively. Thus, the examiner can differentiate between systemic and FF-specific disorders. Therefore, the FF appearance should always be viewed and analyzed in comparison to the surrounding body parts. The clinical examination (CE) of the FF includes an evaluation of the flap's temperature, color change, size and turgor, capillary refill, and bleeding characteristics to fine-needle pin-prick (25). This visual and tactile assessment of FF holds various advantages: It enables fast, inexpensive, and simple to interpret FF monitoring. Furthermore, this practicable method is noninvasive and harmless to both, the patient and the FF. These strengths are reflected in a systematic review revealing FF success rates of ≥95% with CE as exclusive surveillance tool (26). It is, therefore, not surprising that CE is still considered the accepted standard for FF monitoring in an era of advanced and high-tech surveillance strategies (27, 28).

Nonetheless, a caveat of CE is the FF visibility and accessibility, with a limited evaluability of buried FFs or FFs that are difficult to access (e.g., oral cavity) (29). Lighting conditions may distort the FF assessment, as the FF color and appearance depend on the illumination/light source (30). In this context, CE of intraoral, gluteal, and head and neck FF reconstructions are particularly challenging: The complex three-dimensional oral cavity and throat area can limit visual inspection and accessibility. In addition, interindividual FF appearances (e.g., due to age and skin color) and inconsistent warning symptoms of FF failure entail the risk of poor interobserver agreement. In this context, Mofikoya et al. has highlighted the limited validity of CE in Black FF patients (31). The colorimetric evaluation is challenging in this patient population and subtle skin changes may be missed. In elderly patients, morphological age-related variations may hamper the early detection of FF failure in CE. Namely, age spots conceal the actual skin appearance, dermal layers thin out exposing superficial blood vessels, and deep wrinkles complicate adequate turgor assessment (Figure 1) (13, 14, 16, 32). This interindividual heterogeneity of skin appearance may lead to CE-related variance in clinical practice and evaluation, with a lack of standardization and objectification. Varying skill levels and expertise of the assessors can be considered another confounding factor (33, 34). Therefore, easy-to-use and objective tools are needed to quantify and/or objectively determine FF viability.

Previously, complementary methods have been proposed to account for some of CE-related shortcomings. While Urken et al. proposed the exteriorization of a well-vascularized FF segment in buried head and neck defect coverages, van Genechten suggested the use of high color-rendering index, light-emitting diodes to overcome poor lighting during CE of intraoral FFs (30, 35). Such modifications of the rudimentary CE may increase its accuracy and widen its applicability; however, further age-adjusted configurations are required. To date, no standardized protocols for the CE of FF in older patients have been published. As a result, benchmarks for the comparability of CEs in elderly patients undergoing FF surgery are lacking.

### 2.2. Acoustic Doppler sonography

Another adjunct to the basic concept of CE is the use of acoustic Doppler sonography (ADS) as an instrument to sound the blood flow and velocity. Most commonly employed as a handheld device, ADS detects reflected ultrasonic waves (5–8 MHz) and translates these bio-signals into an acoustic feedback (36). Accordingly, the shift in signal quality from a three-phase (tri-or biphasic) to a single-phase (monophasic) arterial response can be an early warning sign of imminent arterial occlusion. In contrast, the initial stages of venous occlusion usually remain unnoticed, whereas in the late stages the arterial signal takes on a “water-hammering” characteristic prior to vanishing (37). The absence of any arterial or venous signal can be deemed advanced vascular blockage and requires immediate intervention (1, 38). Yet, the acoustic differentiation of the microvascular anastomosis from adjacent native vessels requires extensive training. Acoustic coupling/impedance and hard-to-reach flaps are additional obstacles to an accurate assessment of FF.

Despite relatively few clinical studies investigating the efficacy of ADS alone, this noninvasive and recordable method is widespread in FF monitoring (1). The popularity of ADS is likely due to its convenient, fast, and cost-effective application. In addition, the concept of ADS has been established in various other medical settings, underscoring its universal applicability. However, the sensitivity of the ADS mainly concentrates on the detection of blood flow through superficial FF vessels, with poor specificity (38). Given this narrow detection distance, the assessment of blood perfusion in buried flaps is often not possible or reliable. The impact of aging on the detectability of ADS remains to be elucidated: While Marioni et al. and Soria et al. demonstrated inconsistencies in acoustic patterns of ADS among elderly patients, Turrà et al. reported the successful usage of CE and ADS to monitor head and neck FFs in 28 patients aged >60 years and above (39–41).

In sum, ADS has its raison d'être as a valuable adjunct to mere CE and as an initial screening tool for blood flow in the FF. Yet, no definitive conclusions regarding the FF condition can be drawn from ADS alone, rendering the pairing with CE mandatory. Future studies are needed to investigate whether senescence and age-related changes may confound the signals of ADS.

## 3. Modern and innovative alternatives/adjuncts in free flap monitoring

### 3.1. Color duplex ultrasonography

Leveraging the same technology as ADS, color duplex ultrasonography (CDS) is an instrument to visualize ultrasonic waves and thus FF vascularity. FF viability can therefore be assessed acoustically through Doppler outputs, and visually by correlating colors with blood velocities (42). To this end, the microvascular anastomosis is traced *via* a handheld probe, with the perfusion being examined on a viewing monitor (21). Several studies have demonstrated the applicability of CDS as a useful monitoring technique of buried FFs: While Vakharia et al. provided evidence on the usefulness of CDS as a safe and noninvasive monitoring tool in buried facial reanimation FFs, a German study highlighted CDS as a time-efficient and reliable method for the postoperative assessment of vascularized free bone flaps (43, 44). Further, Cuthbert et al. verified the potential of CDS in monitoring a free jejunal flap for oesophageal reconstruction (45). In a comparative study of 45 elderly FF patients (mean age: 66 years), Lethaus et al. concluded that CDS is more precise and reliable than ADS. When localizing FF perforators preoperatively, the authors calculated a sensitivity and positive predictive value of 97.9% and 100% for CDS, respectively (46).

However, these advantages are (partially) countered by costly resources. In order to accurately harness and interpret CDS, an ultrasound technician and a radiologist are needed to take care of the device maintenance/handling and visual analysis, respectively. In addition, the equipment is quoted at up to $225,000 (43). If surgeons are to reliably analyze the diagnostic signals of CDS, they need to undergo thorough training and instruction beforehand. Therefore, this resource-consuming method is more likely to be implemented as selective-complementary than as serial-standard FF monitoring technique.

### 3.2. Flow coupler

The Flow Coupler (FC) technology combines a coupler that is commonly used during anastomosis in FF surgery with an implantable ultrasonic micro-doppler probe. Intraoperatively inserted, the device allows continuous postoperative monitoring of the venous anastomosis, with change or loss of the signal possibly indicating microvascular compromise (21, 47). Zhang et al. highlighted the potential of FC in head and neck FF surgery, reporting an accurate flow signal interpretation in 90% of all cases (48). Furthermore, FC was found to be particularly suitable for the monitoring of buried FFs and difficult-to-reach FFs, such as in oropharyngeal reconstruction cases (49).

In contrast, when comparing FC with a non-flow coupler (including external Doppler monitoring) in 119 patients undergoing abdominal-based breast reconstruction, Kempton et al. shed light on the adverse side effects of FC. Namely, a high false positive rate in intra- and postoperative settings resulted in frequent signal-correcting procedures and significantly more thrombotic events have been documented in FC usage (50). However, these findings should be interpreted with cautions since such FC-related complications could not be confirmed in further studies. Instead, in a 2014 study investigating 220 outcomes of free-flap breast reconstructions, no statistically significant differences were found between an implantable Doppler probe and FC, with comparable false-positive rates and thrombotic events (51). Similarly, while Chadwick et al. highlighted the safe and reliable usage of FC for buried free flap monitoring, Bowe et al. reported no false positives/negatives and a significantly higher flap salvage rate (compared to CE) during their five-year FC experience (52, 53).

These contradictory findings reflect the unclear relevance of FC in postoperative FF surveillance. Given the novelty of FC (FDA approval in 2010), long-term studies are still lacking (51). However, in the FF surgery of elderly patients, FC was found to be beneficial. Investigating the use of FC in 217 FF patients with a mean age of 63 years, Troob et al. reported positive and negative predictive values of 64,3% and 98.9%, respectively (47). FC was able to accurately detect venous thrombosis, while being safe in application and holding the potential to improve FF salvage rates. Despite these promising findings, future large-scale studies are necessary to validate its gerontological efficacy and reliability as well as to identify possible perioperative risks.

### 3.3. Implantable Doppler

First introduced in 1988 by Swartz et al., the implantable Doppler (ID) probe has been established as a valuable FF monitoring technology (54). A piezoelectric crystal embedded in a silicon sheath is attached directly to the microanatomized venous and/or arterial vessel. Connecting wires then transmit the signals to an equivalent of an acoustic Doppler device (21). Hence, the implantable Doppler offers instantaneous and permanent monitoring of the blood flow through the artery and/or vein. Given the direct implantation on the vessel, this method is particularly suitable for the postoperative evaluation of buried FFs (55). Any alteration of the signal's strength and consistency may indicate vascular comprise (54). Theoretically, such immediate feedback on the FF viability allows prompt (surgical) intervention and, therefore, increases the likelihood of FF salvage.

A 2016 meta-analysis corroborated these theoretical considerations: Han et al. demonstrated that ID was associated with significantly better rates of FF success and salvaging when compared with conventional clinical monitoring methods (56). However, the same meta-analysis also revealed an ID-related false-positive rate of up to 17%. This is in line with a case series of 74 pharyngoesophageal and tracheal reconstructions, in which ID generated 31% of false-positive rates (24). The lack of ID-related specificity resulted in unnecessary surgical exploration. Such concerns about ID malfunctions and the susceptibility to false-positive signals were also raised in a study published in 2021 by Pier et al. (57). Specifically, the authors highlighted the detrimental nature of false-positive ID signal loss and reported that one-third of ID malfunctions were associated with patient complications.

Interestingly, an in-depth analysis by Schmulder et al. demonstrated that the value of ID varies by subspecialty (58). While ID was found to be particularly effective in FF monitoring of head and neck and breast reconstructions, trauma/orthopedics specialties benefited less from this technology. The special suitability of ID for postoperative assessment of FFs in the head and neck region was verified in a recent study. In a retrospective analysis of 65 cases with different FF types and locations across the head and neck area, Dunklebarger et al. concluded that ID was highly effective for the surveillance of buried FFs (55). Furthermore, a meta-analysis investigating the diagnostic test precision suggested that arteries can be more accurately evaluated than veins *via* ID (59). A cost-effectiveness analysis reflects these pros and cons regarding the use of ID: While Poder et Fortier confirmed the effectiveness of ID, they also indicated excess costs of nearly EUR 100 per patient (compared to conventional methods) (60).

In sum, ID holds promising potential as an innovative FF monitoring tool with significantly higher FF salvages rates than CE (61). Kim et al. have demonstrated the successful application of real-time FF monitoring *via* wireless Wi-Fi technology (62). This concept enabled simultaneous and convenient FF surveillance at any time and in any situation, with high sensitivity and specificity. However, regardless of the novelty and the advantages of ID, institutions planning to adopt ID should also account for its drawbacks such as the high incidence of false-positive signals and surcharges. In this context, it is noteworthy, that in a matched case-control study investigating the utility of ID among patients with a mean age of 60 years, ID's effectiveness and potential to increase FF success were reaffirmed (63). This finding suggests that ID could also be beneficial among elderly patients.

### 3.4. Laser Doppler flowmetry

Laser Doppler Flowmetry (LDF) is another noninvasive monitoring technique for microcirculation in FFs. LDF also exploits the Doppler effect, with light-tissue interactions as the underlying biotechnical principle (64). A light source emits laser signals that are scattered and reflected by the FF. These reflections are subsequently detected by an optical fiber (65). Strikingly, circulating blood cells create—depending on the velocity of blood flow—a characteristic pattern (that differs from that of non-moving tissue) (38). Based on this pattern, information about the relative value of blood flow can be derived; absolute and exact numbers cannot be measured *via* LDF (38).

Several studies have advocated the use of LDF as FF monitoring technique, emphasizing its real-time measurement, accuracy, and objectivity (38, 66–69). Yuen and Feng reported their five-year experience using LDF in 232 microvascular composite-tissue transfers: LDF detected vascular comprise in all cases, with no false positive or negative misinterpretations. Even more, the flap viability was nearly 100%. In addition, LDF may help differentiate arterial from venous occlusion in free tissue transfer (65).

Researchers report mainly a pragmatic-economic downside of this technology. Expensive start-up and maintenance costs relativize the aforementioned advantages (38). Accordingly, in more recent years, the number of studies investigating and recommending the use of LDF is decreasing. Further, using LDF, Zhang et al. provided evidence that age has a remarkable impact on blood flow (70). LDF-related reference ranges, scores, and relative flow parameters may differ significantly among elderly patients. However, to date, standardized LDF norm values and comparison charts for older patients are lacking. Despite its limitations, LDF may be considered a valuable adjunct in the detection of suspected FF failure and as a validation tool. Of note, LDF has also been studied in combination with tissue spectroscopy/spectrometry. Both technologies apparently complement each other favorably, with promising results in reliable prediction of ischemia in FFs (71–73).

### 3.5. Near-infrared spectroscopy

Near-infrared spectroscopy (NIRS) is a useful adjunct to LDF in that it allows continuous, noninvasive, and bedside monitoring of FF tissue oxygenation (74). NIRS-based devices measure the selective absorption of near-infrared light by oxygen-dependent tissue chromophores contained in hemoglobin (75). The percentage of saturated hemoglobin (StO_2_) is then computed from the ratio of oxygenated and deoxygenated hemoglobin (74, 76). Indicating the balance between oxygen supply and consumption, StO_2_ is a biomarker for tissue (and FF) oxygenation. As such, StO_2_ also indirectly reflects tissue perfusion/vascularity (76). Therefore, NIRS not only complements other FF monitoring techniques but also functions as a stand-alone instrumental tool for the real-time measurement of fluctuations in FF hemodynamics, thus indicating early detection of vascular compromise (75, 77). NIRS can even detect vascular compromise prior to any clinical manifestation (77).

Given these features, numerous studies accredit NIRS special suitability as a monitoring technique. Namely, versatility, accuracy, and reliability were found to be NIRS' key aspects (74–81). With nearly 100% sensitivity and specificity, NIRS has been found to be an excellent tool for identifying arterial and venous comprise (74, 77, 82). This pinpoint detectability has translated into FF salvage rates of about 90% (75, 77). In addition, NIRS can be used to adequately monitor buried FF—as long as the covering skin thickness does not exceed the penetration range of the NIRS sensor (83). According to Ouyang et al. and Chao et al., depth detection down to 20 millimeters is achievable by NIRS (1, 79). This variety of NIRS advantages is further extended by its user-friendliness (76). Although there are no strict cut-off values and manufacturer-dependent variations, a relative drop (compared with baseline readings) of about 20 points commonly indicates FF perfusion failure (76, 84). Thus, inexperienced nursing staff and junior physicians/residents can take over FF monitoring. Even more, NIRS holds potential for remote FF monitoring, without the need for in-hospital presence (78). These strengths of NIRS are reflected in a recent systematic review: Bian et al. concluded that NIRS “provides superior flap salvage and survival rates compared with [CE], which translates to cost savings and a reduction in workload” (85). As a result, this NIRS concept is particularly exciting for smaller peripheral facilities with limited manpower and may possibly balance the additional IRS-associated costs.

In fact, implementing NIRS as standard technique should not be underestimated financially, with reported device costs up to $30,000 and maximal sensor costs as high as $1,200 (76). Notably, after performing a comparison of cost-effectiveness between CE alone and NIRS, Schoenbrunner et al. drew surprising conclusions (86): The complementary use of NIRS was found to increase the effectiveness minimally and thus, CE alone represented the more cost-effective FF monitoring option. Along with the debatable cost-effectiveness, there is the question of how susceptible NIRS measurements are across a diverse FF patient population. Indeed, Salgarello et al. identified several variables that correlate significantly with regional oxygen saturation (81). Besides FF size and skin FF area, the patient BMI also markedly affected NIRS oximetry data. The assumption that age also has a relevant impact on NIRS measurements is, therefore, plausible but has not yet been investigated. Such an age-specific impact may shift or invalidate the currently applied NIRS interpretation thresholds. Takasu et al. has provided first evidence that NIRS may also be used effectively in elderly patients (87): More specifically, in two 63-year-old patients, rapidly dropping StO_2_ values triggered the FF re-exploration—at an early stage and prior to any clinical manifestation. Thus, venous occlusion could be promptly diagnosed, with both flaps being salvaged *via* re-anastomization. Yet, future studies defining the relevance of senescence are needed to conclusively assess the usefulness of NIRS in elderly patients. Otherwise, NIRS is a modern and promising FF monitoring technique that offers a variety of benefits and may—after robust validation—find more widespread acceptance.

### 3.6. Hyperspectral imaging

Hyperspectral Imaging (HSI) is a fast and noninvasive and nonionizing monitoring technique based on spectrometric tissue analysis (88–90). The target tissue is illuminated by halogen lamps and remitted light is detected in a wavelength spectrum from visual to near-infrared light (380–1000 nm) (91). Due to the heterogeneity of the tissue, distinct remission spectra are generated and subsequently saved as a three-dimensional data cube (92). Built-in software tools then process the data into high-resolution color-coded images, with four computed tissue parameters (76, 92): While the hemoglobin oxygenation (StO_2_) and the near-infrared perfusion index (NIR) report the surface hemoglobin oxygen saturation, the distributions of hemoglobin and water are depicted in form of the tissue hemoglobin index (THI) and the tissue water index (TWI), respectively. As a result, HSI provides precise, objective, and reproducible information on FF viability. In a recent systematic review, NIRS and HSI as two innovative FF technologies were compared (76). While both methods were found to yield comparable effectiveness, Lindelauf et al. also highlighted their technological-methodological differences. HSI does not—unlike NIRS—feature continuous measurement and, thus, does not allow permanent FF surveillance. Any moving or manipulation of the recorded tissue will skew the HSI measurements. However, with HSI being a contactless monitoring technique, imaging can also be performed intraoperatively and in a more patient-comfortable manner.

Due to the novelty of HSI, large-scale validation studies and robust clinical data are lacking so far. However, the studies conducted to date share a consensus: HSI is an exceptionally promising option for FF monitoring. The unique information value derived from the four HSI tissue parameters delivers early evidence of vascular comprise and accurately identifies necrotic FF areas (76, 92–95). In addition, HSI can help distinguish between arterial occlusion (low THI and low StO_2_) and venous congestion (high THI and low StO_2_) (93). In a prospective observational cohort study with 22 patients undergoing FF surgery for soft tissue reconstruction, Kohler et al. were able to reproduce these HSI-linked benefits. These findings even encouraged the authors to claim a “superiority [of HSI] to clinical and Doppler ultrasound monitoring assessments” (96). Tiehm et al. also joined this chorus of optimism, with the observation of HSI revealing FF malperfusion nearly 5 h earlier than CE (95).

In contrast, HSI is criticized for its reliance on ambient lighting (95, 97). To prevent a loss of data quality, interference/background light should be minimized during imaging. Further, its cost-effectiveness and long-term practicability remain to be elucidated (96). HSI-related expenses, however, mainly include the one-time purchase of the device (about $40,000); ongoing costs are rarely incurred (76, 92). Regarding the applicability of HSI in elderly patients, the necessary resting posture during recording (for about 15 s) may be a potential problem. With many old patients suffering from tremor and restlessness, HSI images may end up blurred and invalid. This limitation was also reported by Courtenay et al. who recently tested the HSI method in 115 elderly patients with non-melanoma skin cancer (98). The authors also noted a lack of comfort among elderly patients. This HIS-associated handicap could be overcome, for example, by ergonomic support aids or platforms. Nevertheless, future research is needed to both broadly validate HSI as FF monitoring tool and to identify age-specific challenges.

## 4. Further free flap monitoring methods and their applicability in elderly patients

(i) Thermal Imaging (TI) is a novel and noninvasive modality to detect vascular perfusion. By measuring radiation (which is proportional to the body temperature) and converting it into visible images, TI creates heat maps (13, 99). With the body temperature correlating to the blood flow, TI can be considered a surrogate detector of FF vascularity. Frohwitter et al. reported the successful application of TI in a 90-year-old woman who underwent microvascular radial forearm flap reconstruction (13). The temperature dynamics visualized by TI provided valuable insights into the rheology of FF, both intraoperatively and postoperatively.
(ii) Oxygen partial pressure (PtO_2_) measurement within the FF represents another promising apparative monitoring technique. In this context, polarographic micro-catheter probes surgically implanted in the FF provide continuous electrochemical signals, with marked decreases in PtO_2_ indicating FF failure (100, 101). Depending on where the PtO_2_-sensing needle tip is placed, this technique allows measurements in variable tissue layers. One shortcoming of most of these needle probes is a single point and, thus, very selective PtO_2_ measurement within the tissue. Ashkenazi et al. managed to overcome this issue, by developing a PtO_2_ sensing probe, that enables multiple measurements along the probe shaft (102). The underlying technology is based on a side-firing and light-collecting optical fiber tip placed in an oxygen-sensitive dye-coated glass capillary. This way PtO2 scans at multiple tissue depths can be recorded at the same time, avoiding the need for repeated needle translocations and resulting tissue damage. In 2013, a German study investigated the use of PtO_2_ surveillance in 125 microvascular FF cases (100). The polarographic technology indicated the necessity of salvage surgery at an early stage and error-free. This finding is particularly relevant given the average patient age of over 60 years, which underscores the instrument's efficacy in an elderly patient population. Similarly, when testing PtO_2_ monitoring during the surgical after-care of 21 elderly FF patients (mean age: 61 years), Trignano et al. also reported promising results (103). Alarming PtO_2_ values accurately triggered the re-exploration of three FFs, one of which could be salvaged. More recently, Dejean et al. reported the successful use of this PtO_2_ technology in the postoperative surveillance of elderly women who underwent FF breast reconstruction (104). Nonetheless, this method has been rarely used and clinicians insist on the importance of parallel periodic clinical FF examinations (103, 105, 106).
(iii) As mentioned above, in cases of hard-to-reach and buried flaps, monitoring *via* skin paddles may represent a valuable adjunct to CE. Recently, two case series have documented the usefulness of such cutaneous paddles as a monitoring strategy also in elderly FF patients. When evaluating suprastomal skin paddle monitoring after FF reconstructions of laryngopharyngectomies, Revenaugh et al. found relatively few complication rates and low false-positive rates (107). In addition, this method allowed direct monitoring and did not interfere with speech and swallowing functions. These findings are all the more significant in view of the vulnerable patient cohort, with a mean age of 62 years and a history of carcinoma. The skin paddle method has also been shown to be effective in scalp reconstruction: Park et al. reported significantly higher FF salvage rates and total FF survival when compared to conventional monitoring strategies (108). Again, the average patient was older than 62 years and the etiology was predominantly tumor-related. Taken together, the externalization of a skin paddle seems to be a safe and reliable method for FF monitoring, even in advanced patient age. However, both research groups stress the need for compatibility of vascular geometry, without undue functional and aesthetic curtailment.

Further methods, such as the use of indocyanine green dye angiography, spatial frequency domain imaging, carbon dioxide monitoring, or microdialysis have been tested in small-scale studies and/or exclusively in young/healthy patients and, therefore, fall beyond the scope of this review.

## 5. Conclusion

Free flap monitoring is crucial to ensure early detection of vascular comprise and was shown to improve salvage rates. Elderly surgical patients represent a particularly vulnerable population due to an age-related decline in health, rendering free flap surveillance all the more important. Yet, the demands for the ideal monitoring method are high and range from accuracy and reliability through reproducibility and objectivity to cost-effectiveness and user-friendliness. In search of such panacea, numerous monitoring tools and techniques have been proposed, each with its inherent strengths and limitations. In fact, despite promising potential, none of these emerging methods has yet been accepted on a widespread front. In an era of high-tech medicine, the basic clinical examination in combination with Doppler sonography still represents the gold standard although other technologies such as near-infrared spectroscopy are increasingly used in clinical practice. However, advancing digitization holds the potential to compensate for the downsides of clinical assessment (subjective, poorly reproducible, manpower-dependent). Thus, in the future, original manpower may be (even more) replaced by a finely balanced and efficient interaction of man and machine.
